# Melarsoprol Sensitivity Profile of *Trypanosoma brucei gambiense* Isolates from Cured and Relapsed Sleeping Sickness Patients from the Democratic Republic of the Congo

**DOI:** 10.1371/journal.pntd.0003212

**Published:** 2014-10-02

**Authors:** Patient Pyana Pati, Nick Van Reet, Dieudonné Mumba Ngoyi, Ipos Ngay Lukusa, Stomy Karhemere Bin Shamamba, Philippe Büscher

**Affiliations:** 1 Département de Parasitologie, Institut National de Recherche Biomédicale, Kinshasa Gombe, Democratic Republic of the Congo; 2 Department of Biomedical Sciences, Institute of Tropical Medicine, Antwerp, Belgium; Foundation for Innovative New Diagnostics (FIND), Switzerland

## Abstract

**Background:**

Sleeping sickness caused by *Trypanosoma brucei* (*T.b*.) *gambiense* constitutes a serious health problem in sub-Sahara Africa. In some foci, alarmingly high relapse rates were observed in patients treated with melarsoprol, which used to be the first line treatment for patients in the neurological disease stage. Particularly problematic was the situation in Mbuji-Mayi, East Kasai Province in the Democratic Republic of the Congo with a 57% relapse rate compared to a 5% relapse rate in Masi-Manimba, Bandundu Province. The present study aimed at investigating the mechanisms underlying the high relapse rate in Mbuji-Mayi using an extended collection of recently isolated *T.b. gambiense* strains from Mbuji-Mayi and from Masi-Manimba.

**Methodology/Principal Findings:**

Forty five *T.b. gambiense* strains were used. Forty one were isolated from patients that were cured or relapsed after melarsoprol treatment in Mbuji-Mayi. *In vivo* drug sensitivity tests provide evidence of reduced melarsoprol sensitivity in these strains. This reduced melarsoprol sensitivity was not attributable to mutations in *TbAT1*. However, in all these strains, irrespective of the patient treatment outcome, the two aquaglyceroporin (*AQP*) 2 and 3 genes are replaced by chimeric *AQP2/3* genes that may be associated with resistance to pentamidine and melarsoprol. The 4 *T.b. gambiense* strains isolated in Masi-Manimba contain both wild-type *AQP2* and a different chimeric *AQP2*/3. These findings suggest that the reduced *in vivo* melarsoprol sensitivity of the Mbuji-Mayi strains and the high relapse rates in that sleeping sickness focus are caused by mutations in the *AQP2/AQP3* locus and not by mutations in *TbAT1*.

**Conclusions/Significance:**

We conclude that mutations in the *TbAQP2/3* locus of the local *T.b. gambiense* strains may explain the high melarsoprol relapse rates in the Mbuji-Mayi focus but other factors must also be involved in the treatment outcome of individual patients.

## Author Summary

Sleeping sickness, or human African trypanosomosis, constitutes a serious health problem in sub-Sahara Africa. Treatment is a key factor in the control of this disease, not only to save the lives of the individual patients but also to stop transmission. As for other infectious diseases, drug resistance forms a constant threat for sleeping sickness control. Understanding the mechanisms underlying drug resistance is of uppermost importance for evidence-based adaptation of treatment protocols as well as for the development of new drugs. In this study we investigated the phenotype and genotype of more than 40 recently isolated strains of *Trypanosoma brucei gambiense*, the parasite that causes sleeping sickness in West and Central Africa. By comparing the parasites from cured and from relapsing patients in two separated sleeping sickness foci, we can explain the difference in treatment failure rates between these two sleeping sickness foci in D.R. Congo. We also provide evidence that treatment outcome in the individual patient is not exclusively defined by the drug resistance genotype of the infecting parasite but that other factors must be involved.

## Introduction

Human African trypanosomosis (HAT) is a parasitic disease transmitted by tsetse flies (*Glossina sp*) and caused by *Trypanosoma brucei (T.b.) gambiense* and *T.b. rhodesiense*. This disease constitutes a serious public health problem in sub-Saharan Africa, particularly in central African countries like the Democratic Republic of the Congo (DRC), Central African Republic (CAR), the Republic of the Congo and the Republic of South Sudan [1]. The disease evolves from an early stage with trypanosomes invading blood, lymph and peripheral tissues towards the late or neurological stage with invasion of the brain. Chemotherapy of HAT relies on five drugs (eflornithine, melarsoprol, nifurtimox, pentamidine and suramin). Early-stage HAT is treated with pentamidine (*T.b. gambiense*) or suramin (*T.b. rhodesiense*). For treatment of the neurological stage, drugs that are able to pass the blood-brain-barrier, such as melarsoprol, nifurtimox or eflornithine, are necessary [2]. Until recently, melarsoprol was the first line treatment of late stage *gambiense* and *rhodesiense* HAT but for *gambiense* HAT, the nifurtimox-eflornithine combination therapy (NECT) is now recommended by the World Health Organization as first line treatment [3]. This recommendation follows the observation that NECT is as effective as melarsoprol monotherapy with less severe side effects and that NECT is able to cure patients that experienced a relapse after treatment with melarosprol monotherapy [4]. Traditionally, 5–10% of *gambiense* HAT patients treated with melarsoprol could not be cured but in the last decade up to >50% relapse rates were reported in Angola, Central African Republic, Democratic Republic of the Congo, Republic of South Sudan and Uganda [5]–[11]. Particularly problematic was the situation in the HAT focus of Mbuji-Mayi, East Kasai Province in DRC, where a 57% failure rate was observed in patients treated with the 10 days abridged melarsoprol regimen [8]. Various studies have been conducted to explain these unexpectedly high relapse rates, considering either the parasite or the human host being responsible for this phenomenon. Although known mechanisms may be involved, such as mutations of the P2 adenosine transporters and of aquaglyceroporin transporters in the trypanosome membrane, the explanation for treatment failure is probably more complex, including parameters of the parasite (reduced drug sensitivity and higher tissue tropism), the drug (content of active principle and correct administration) and the host including individual differences in pharmacokinetics, co-infections and disease stage [9], [12]–[24].

To investigate the mechanism underlying the high failure rates in the Mbuji-Mayi focus, Pyana and co-workers undertook the large scale isolation of the trypanosome from HAT patients in that focus [25]. Thus, we established a collection of 85 *T.b. gambiense* type I strains from cured and relapsed patients, some of which from the same patient before treatment and after relapse. Among these strains, 41 were adapted to *Mus musculus* in order to test their sensitivity to melarsoprol in an *in vivo* mouse infection model and to analyse some genetic features that may be related to reduced sensitivity to melarsoprol. Recently, some of them were shown to be resistant to pentamidine and melarsoprol by *in vitro* drug sensitivity testing and to carry a *TbAQP*2/3 chimera that was supposed to lead to a reduced uptake of pentamidine in the trypanosome flagellar pocket [20]. In this study, we aimed at 1° investigating the *in vivo* melarsoprol sensitivity phenotype of all 41 mouse adapted *T.b. gambiense* strains isolated in the Mbuji-Mayi focus, 2° investigating some of their genotypic characteristics and comparing them with 4 strains isolated from a sleeping focus with low relapse rates in Masi-Manimba, and 3° relating their phenotype and genotype with treatment outcome of the patients from whom they were isolated.

## Materials and Methods

### Ethics statement

The study in mice was approved by the Veterinary Ethics Committee of the Institute of Tropical Medicine, Antwerp, Belgium (protocol PAR-022) and adhere to the European Commission Recommendation on guidelines for the accommodation and care of animals used for experimental and other scientific purposes (18 June 2007, 2007/526/EG) and the Belgian National law on the protection of animals under experiment.

The parasite strains included in this study belong to the cryobank of the World Health Collaboration Center for Research and Training on Human African Trypanosomiasis Diagnostics at the Institute of Tropical Medicine in Antwerp, Belgium. Their isolation and use for research purposes was approved by the Ethical Committee of the Institute of Tropical Medicine (04441472) and of the Ministry of Health of DRC [25]. The strains are anonymized by using international codes and alias names.

### Trypanosome strains

A list of the *T.b. gambiense* strains from the Mbuji-Mayi focus in East Kasai Province, DRC, is given in Table 1. The isolation history of these strains is described elsewhere [25]. The alias name of each strain indicates whether it was isolated before treatment (BT) or after treatment (AT). Eleven strains were isolated from patients that were cured after melarsoprol treatment. Thirty strains were isolated from patients that relapsed after treatment. Among these 30 strains, twenty belong to “couples”, i.e. isolated from the same patients, before treatment and after relapse. The melarsoprol sensitive and resistant strains included as reference in the *in vivo* drug sensitivity experiment, *T.b. brucei* 427 wild type and *T.b. brucei* 427 AT1/P2 KO (P2 adenosine transporter knock out) were received from the Swiss Tropical and Public Health Institute. For the genotype analysis, 7 extra *T.b. gambiense* type I strains were added (Table 1). Four of these strains were isolated in 2011 from cured patients in the Masi-Manimba focus, Bandundu Province, RDC where high relapse rates after melarsoprol treatment were never observed. Three strains are “old” isolates. LiTat 1.3 is a cloned population of the Eliane strain, isolated in Côte d'Ivoire, and previously shown to be sensitive to melarsoprol and pentamidine *in vitro*, while MBA and KEMLO are two Congolese strains with unknown drug sensitivity profile [26]. All the strains were kept as 250 µl cryostabilates in liquid nitrogen.

**Table 1:** List of *T.b. gambiense* strains used in this study.

HAT focus, country	International code or name	Alias name or clone	Treatment outcome	Couple
Mbuji-Mayi, DRC	MHOM/CD/INRB/2008/56	15BT	cure	
	MHOM/CD/INRB/2008/46	19BT	cure	
	MHOM/CD/INRB/2008/65	29BT	cure	
	MHOM/CD/INRB/2006/13	40BT	relapse	1
	MHOM/CD/INRB/2006/07	40AT	relapse	1
	MHOM/CD/INRB/2006/01	45BT	cure	
	MHOM/CD/INRB/2008/52	48BT	relapse	
	MHOM/CD/INRB/2007/28	57AT	relapse	
	MHOM/CD/INRB/2008/62	85BT	cure	
	MHOM/CD/INRB/2006/09	93AT	relapse	
	MHOM/CD/INRB/2008/63	95BT	cure	
	MHOM/CD/INRB/2008/42	99BT	cure	
	MHOM/CD/INRB/2008/49	104BT	relapse	2
	MHOM/CD/INRB/2008/53A	104AT	relapse	2
	MHOM/CD/INRB/2008/50	105BT	relapse	
	MHOM/CD/INRB/2007/25B	108AT	relapse	3
	MHOM/CD/INRB/2007/27	108BT	relapse	3
	MHOM/CD/INRB/2008/60	113AT	relapse	4
	MHOM/CD/INRB/2008/45	113BT	relapse	4
	MHOM/CD/INRB/2006/11A	116AT	relapse	
	MHOM/CD/STI/2006/02	130BT	relapse	
	MHOM/CD/INRB/2008/64	141BT	cure	
	MHOM/CD/INRB/2005/02A	146BT	relapse	5
	MHOM/CD/INRB/2006/05	146AT	relapse	5
	MHOM/CD/INRB/2007/26A	147AT	relapse	
	MHOM/CD/INRB/2005/01B	148BT	relapse	6
	MHOM/CD/INRB/2006/14	148AT	relapse	6
	MHOM/CD/INRB/2006/06A	163AT	relapse	
	MHOM/CD/INRB/2008/47	167BT	relapse	7
	MHOM/CD/INRB/2008/43	167AT	relapse	7
	MHOM/CD/INRB/2008/59	174BT	relapse	8
	MHOM/CD/INRB/2007/29	174AT	relapse	8
	MHOM/CD/INRB/2008/37A	186BT	cure	
	MHOM/CD/INRB/2006/12A	223AT	relapse	
	MHOM/CD/INRB/2006/21B	340AT	relapse	
	MHOM/CD/INRB/2007/22B	346BT	relapse	9
	MHOM/CD/INRB/2007/24B	346AT	relapse	9
	MHOM/CD/INRB/2006/23A	348BT	cure	
	MHOM/CD/INRB/2006/16	349BT	relapse	10
	MHOM/CD/INRB/2006/19	349AT	relapse	10
	MHOM/CD/INRB/2007/34	378BT	cure	
Masi-Manimba, DRC	MHOM/CD/INRB/2011/01	MM01	cure	
	MHOM/CD/INRB/2011/03	MM03	cure	
	MHOM/CD/INRB/2011/05	MM05	cure	
	MHOM/CD/INRB/2011/06	MM06	cure	
Kinshasa, DRC	MAN/ZR/74/ITMAP1811	MBA	unknown	
Bwamanda, DRC	MAN/ZR/74/ITMAP1821	KEMLO	unknown	
Daloa, Côte d'Ivoire	ELIANE	LiTat 1.3	unknown	

In alias name: AT = after treatment, BT = before treatment. Treatment outcome: outcome of patient treated with melarsoprol (in Mbuji-Mayi) or with nifurtimox-eflornithine combination therapy (Masi-Manimba). Couple = number of the couple of two strains isolated from the same patient.

### Expansion of parasite populations

Each stabilate was thawed in a water bath at 37°C and immediately, 250 µl of phosphate buffered saline glucose (PSG, 7.5 g/l Na_2_ HPO_4_ 2H_2_O, 0.34 g/l NaH_2_ PO_4_ H_2_O, 2.12 g/l NaCl, 10 g/l D-glucose, pH 8) were added. This mixture was kept on ice until inoculated intraperitoneally (IP) into two 1–2 months old female OF-1 mice (Charles River, Belgium). Two days before infection, these mice had been immunosuppressed by IP injection with 200 mg/kg body weight (BW) of cyclophosphamide diluted in water (Endoxan, Baxter, Lessing, Belgium). Parasitaemia was monitored three times a week on a fresh preparation of 5 µl of tail blood according to the matching method of Herbert and Lumsden (1976). If needed, immunosuppression was repeated after 5 days, until the parasitaemia reached 10^7.5^/ml or more and the trypanosome population was large enough to inoculate 12 mice for the *in vivo* melarsoprol sensitivity experiment. For the inoculation of the mice in the *in vivo* drug sensitivity experiments (see below), 30 to 40 µl of infected tail blood was diluted in about 3 ml of PSG, trypanosomes were counted in a Uriglass cell counting chamber (Menarini Diagnostics) and the suspension was further diluted in PSG to obtain a concentration of 250 trypanosomes/µl.

### *In vivo* melarsoprol sensitivity experiments

For the treatment of mice, melarsoprol (Aventis, 5 ml vials of 180 mg in propylene glycol, lot nr. 725) was freshly diluted in 50% polyethylene glycol (PEG_400_, Sigma Aldrich, Belgium) to concentrations of 1 mg/ml.

For each experiment, twelve female OF-1 mice (1–2 months old, 20–30 g body weight (BW)) were immunosuppressed as described above, two days before inoculation and subsequently at days 3, 11, 25 and 85 post-infection (DPI). Each mouse was inoculated IP with 200 µl PSG containing 5×10^4^ trypanosomes. Parasitaemia was monitored as described above from day 4 post-infection onwards. As soon as trypanosomes were detected in all mice (between 4 and 6 DPI), one group of 6 mice received IP injections of melarsoprol at 10 or 12 mg/kg BW during 4 consecutive days. On day 5–10 post infection, the mice of the control group were euthanised with an IP injection of sodium pentobarbital at 350 mg/kg BW (Nembutal, CEVA Santé Animale, Brussels, Belgium) and blood was taken by heart puncture on heparin to prepare sediments of pure trypanosomes for DNA extraction and genetic analysis (see below). The mice of the melarsoprol treated group were checked for the presence of trypanosomes in tail blood two times a week during the first two weeks and subsequently once a week for maximum 100 days. As soon as a trypanosome was detected in at least one mouse of this group, the experiment was stopped and the strain was considered “resistant”. Relapsing mice were sacrificed and blood was collected on heparin to separate the trypanosomes from the blood via DEAE chromatography for further genotypic analysis. On days 90 to 100 postinfection, all mice that remained trypanosome negative were sacrificed and blood was collected on heparin where after it was run over two mini Anion Exchange Centrifugation Technique columns to detect subpatent parasitaemia [27].

### Trypanosome purification and genomic DNA extraction

Infected blood from mice was passed over a DEAE cellulose column (1∶6 blood∶gel ratio) to separate the trypanosomes from the blood [28]. The trypanosomes eluting from the column were washed three times with 5 ml ice-cold PSG by centrifugation. After the last centrifugation, the supernatant PSG was discarded and the trypanosome sediment was frozen at −80°C until DNA extraction. After thawing and addition of 200 µl of phosphate buffered saline, pH 8, genomic DNA was extracted from the trypanosome sediment with the Maxwell 16 Tissue DNA Purification kit on the Maxwell 16 robot (Promega, Madison, WI, USA) and DNA was stored at −20°C. DNA concentrations were measured with the Nanodrop ND-1000 UV-Vis spectrophotometer (NanoDrop Technologies, Wilmington, USA) and adjusted to 10 ng/µl if appropriate.

### Genotyping

#### mlSfaNI PCR-RFLP for TbAT1

The protocol to amplify a 677-bp fragment of the *TbAT1* gene and to digest it with SfaNI was based on Mäser *et al.* [17]. A 20 µl reaction volume contained 1× PCR buffer (Qiagen, Venlo, The Netherlands), 1 mM MgCl_2_ (Qiagen), 200 µM of each dNTP (Eurogentec, Seraing, Belgium), 0.8 µM of Sfa-s (5′-CGCCGCACTCATCGCCCCGTT-3)′ and Sfa-as (5′-CCACCGCGGTGAGACGTGTA-3′) (Biolegio, Nijmegen, The Netherlands), 0.4 U Hotstart *Taq* plus polymerase (Qiagen), 0.1 mg/ml acetylated BSA (Promega) and 2 µl target DNA. Amplification condition were as follows: initial denaturation at 94°C for 5 min, 30 cycles 1 min at 94°C, 1 min at 65°C and 2 min at 72°C and final extension at 72°C for 10 min. Five µl of the PCR products were digested 16 hours at 37°C in a 10 µl reaction mixture containing 1× NEB 3.1 buffer with 2 U of SfaNI restriction enzyme (New England Biolabs, Hitchin, UK), followed by 20 minutes incubation at 65°C. Differential banding was analysed using 10 µl on a 2% agarose gel run for 30 min at 135 V and stained with ethidium bromide.

#### PCR for full-length TbAT1

The protocol to amplify the full length *TbAT1* gene and its flanking regions was slightly modified from Graf et al. [20]. A 20 µl reaction volume contained 1× Coral Load Buffer, 1 mM MgCl_2_ (Qiagen), 200 µM of each dNTP (Eurogentec), 0.8 µM of TbAT1_F (5′-GAAATCCCCGTCTTTTCTCAC-3)′ and TbAT1_R_Tbg (5′-ATGTGCTGACCCATTTTCCTT-3)′ primers (Biolegio), 0.4 U Hotstart *Taq* plus polymerase (Qiagen), 0.1 mg/ml acetylated BSA (Promega) and 2 µl target DNA. Amplification conditions were: initial denaturation at 95°C for 5 min, 24 cycles of 1 min at 95°C followed by 1 min at 56°C and 2 min at 72°C, final extension at 72°C for 5 min. Ten µl of the PCR product were electrophorised in a 2% agarose gel for 30 min at 135 V and stained with ethidium bromide for detection of the amplicons. For selected strains, the amplicons were cleaned up and concentrated using a PCR clean-up kit (Qiagen) and sent out for bidirectional direct sequencing at the VIB Genetic Sequencing Facility (Antwerp, Belgium) using the described PCR primers and additional internal primers to cover *TbAT1*.

##### Locus specific PCR for aquaglyceroporin genes

The protocols to amplify either the *AQP2* locus or the combined *AQP2* and *AQP3* locus, including the AQP2/3 chimera were slightly modified from Graf et al. [20]. To amplify the wild-type *APQ2* locus, a 20 µl reaction volume contained 1× Coral Load Buffer (Qiagen), 1 mM MgCl_2_ (Qiagen), 200 µM of each dNTP (Eurogentec), 0.8 µM of AQP2/3_F 5′-AAGAAGGCTGAAACTCCACTTG-3′ and AQP2_R 5′-CTTCGGGAGAAACAAAACCTC -3′ primers (Biolegio), 0.4 U Hotstart *Taq* plus polymerase (Qiagen), 0.1 mg/ml acetylated BSA (Promega) and 2 µL target DNA. Amplification conditions were: initial denaturation at 95°C for 5 min, 24 cycles of 1 min at 95°C followed by 1 min at 60°C and 2 min at 72°C, final extension at 72°C for 10 min. Ten µl of the PCR product were electrophorised in a 2% agarose gel for 30 min at 135 V and stained with ethidium bromide for detection of the amplicons. To amplify the combined *APQ2* and *APQ3* locus, a 20 µl reaction volume contained 1× Coral Load Buffer (Qiagen), 1 mM MgCl_2_ (Qiagen), 200 µM of each dNTP (Eurogentec), 0.8 µM of AQP2/3_F 5′-AAGAAGGCTGAAACTCCACTTG-3′ and AQP2/3_R 5′-TGCACTCAAAAACAGGAAAAGA-3′ primers (Biolegio), 0.4 U Hotstart *Taq* plus polymerase (Qiagen), 0.1 mg/ml acetylated BSA (Promega) and 2 µL target DNA. Amplification conditions were: initial denaturation at 95°C for 5 min, 24 cycles of 1 min at 95°C followed by 1 min at 60°C and 4 min at 72°C, final extension at 72°C for 10 min. Ten µl of the PCR product were electrophorised in a 0.8% agarose gel for 30 min at 135 V and stained with ethidium bromide for detection of the amplicons. For selected strains, amplicons were cleaned up and concentrated using a PCR clean-up kit (Qiagen) and sent out for bidirectional direct sequencing at the VIB Genetic Sequencing Facility (Antwerp, Belgium) using the described PCR primers and additional internal primers to cover *TbAQP2*.

##### Cloning of aquaglyceroporin variants

The full-length ORFs of the *AQP2* and *AQP2/3* genes were amplified from genomic DNA using a proofreading polymerase (Phusion High-Fidelity DNA Polymerase, Thermo Scientific, Waltham, MA, USA). All cloning primers (Biolegio) contained a 5′ extension of 15 nucleotides from the trypanosomal expression vector pHD309, as required for the In-Fusion Cloning reaction (Clontech, Takara Bio, Otsu, Japan) [29]. The primer sets consisted of a forward primer (IF HindIII 5′-AACTGCAACG *AAGCTT* ATGCAGAGCCAACCAGACA-3′) and an *AQP2* specific reverse primer (IF BamHI AQP2 5′-TAAATGGGCA *GGATCC* TTAGTGTGGAAGAAAATATTTGTACAG-3′) for amplification of *T.b. gambiense* MM01, LiTaR1 and MBA or an *AQP2/*3 specific reverse primer (IF BamHI AQP2/3 5′-TAAATGGGCA *GGATCC* TTAGTGTGGCACAAAATATTTGTACA-3′) for amplification of *T.b. gambiense* 348BT. Plasmids were purified from several transformed *E. coli* and sent out for bidirectional direct sequencing at the VIB Genetic Sequencing Facility (Antwerp, Belgium).

##### PCR-RFLP for AQP2/3 chimeric alleles from Mbuji-Mayi

A 278-bp fragment was amplified from the predicted *AQP2/3* chimeras from Mbuji-Mayi in a 20 µl reaction volume that contained 1× PCR buffer (Qiagen), 1 mM MgCl_2_ (Qiagen), 200 µM of each dNTP (Eurogentec), 0.4 µM of AQP2-RFLP-F (5′-GAACTCATTTCCACCGCAGT-3)′ and AQP2-RFLP-R (5′-AGTCCAAAGATACCTCCAAACA-3′) (Biolegio), 0.4 U Hotstart *Taq* plus polymerase (Qiagen), 0.1 mg/ml acetylated BSA (Promega) and 2 µl target DNA. Amplification conditions were as follows, initial denaturation at 94°C for 5 min, 24 cycles of 94°C for 30 sec, 60°C for 30 sec and 72°C for 30 sec and final annealing at 72°C for 5 min. Five µl of the PCR products or 1 µg of plasmid DNA, containing a cloned *AQP2/3* variant, were digested for 15 min at 37°C in a 15 µl reaction mixture containing 1 µl FastDigest Green buffer with 1 µl of either AvaI or SduI FastDigest restriction enzymes (Thermo Scientific), followed by 20 minutes inactivation at 80°C. Differential banding was analysed using electrophoresis of 10 µl on a 3% small fragment agarose gel run for 30 min at 135 V and stained with ethidium bromide.

## Results

### Phenotype

In a preparatory experiment, mice were infected with strain 348BT, isolated from a patient who had been cured, and were treated with melarsoprol at 1, 2, 3, 4, 5, 8 and 10 mg/kg BW. The minimum melarsoprol dosage needed to cure the mice from the infection appeared to be 10 mg/kg BW. This dosage was then used to treat mice infected with all the 41 *T.b. gambiense* strains and with the two *T.b. brucei* strains. Some mice infected with *T.b. gambiense* strains 15BT, 163AT and 346AT experienced a relapse that was detectable only about three months after the infection and after immunosuppression with cyclophosphamide (Table 2). All mice infected with the other *T.b. gambiense* strains and with both *T.b. brucei* strains (including the AT1/P2 KO strain) got cured by melarsoprol at 10 mg/kg/BW, as defined by the absence of detectable relapse up to 100 days after infection. To confirm the apparent melarsoprol resistance of 15BT, 163AT and 346AT, the experiment was repeated with these strains and with melarsoprol treatment at 10 and 12 mg/kg BW. All mice infected with 15BT remained without detectable parasitaemia after treatment. Among the mice infected with 163AT, one mouse relapsed at DPI 28 after treatment with 10 mg/kg BW and one mouse relapsed at DPI 31 after treatment with 12 mg/kg BW melarsoprol. All mice infected with 346AT and treated with 10 and 12 mg/kg BW melarsoprol, relapsed at DPI 20 (Table 2).

**Table 2:** Phenotype of melarsoprol resistant strains.

	10 mg/kg BW		10 mg/kg BW (rep)		12 mg/kg BW	
Alias name	numbers relapsed	DPI	numbers relapsed	DPI	numbers relapsed	DPI
15BT	2	85*	0	na	0	na
163AT	3	88*	1	28	1	31
346AT	1	90*	6	20	6	20*

Number of relapsing mice (out of 6 infected) and day post-infection that relapses were observed after treatment with melarsoprol at different dosages and repetitions. DPI: days post-infection, BW: body weight, rep: repetition, na: not applicable,

*: relapsing population used for AQP2/3 RFLP analysis.

### Genotype

#### *TbAT1* P2 adenosine transporter gene

**Figure 1:**
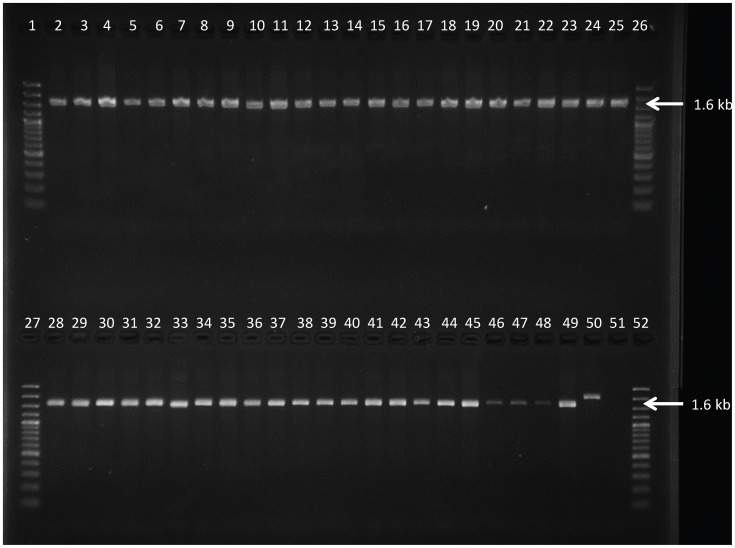
Restriction digest profile generated with SfaNI PCR-RFLP on DNA of the *T.b. gambiense* strains as listed in Table 1 and of the two *T.b. brucei* control strains. Lanes 1, 26, 27 and 52 = GeneRuler 100 bp Plus DNA Ladder (Fermentas), lanes 2 to 44 = *T.b. gambiense* strains isolated from Mbuji-Mayi, lanes 45–48 = *T.b. gambiense* strains isolated from Masi-Manimba, lane 49 = *T.b. brucei* 427 WT, lane 50 = *T.b. brucei* 427 AT1/P2 KO, lane 51 = negative PCR control.

**Figure 2:**
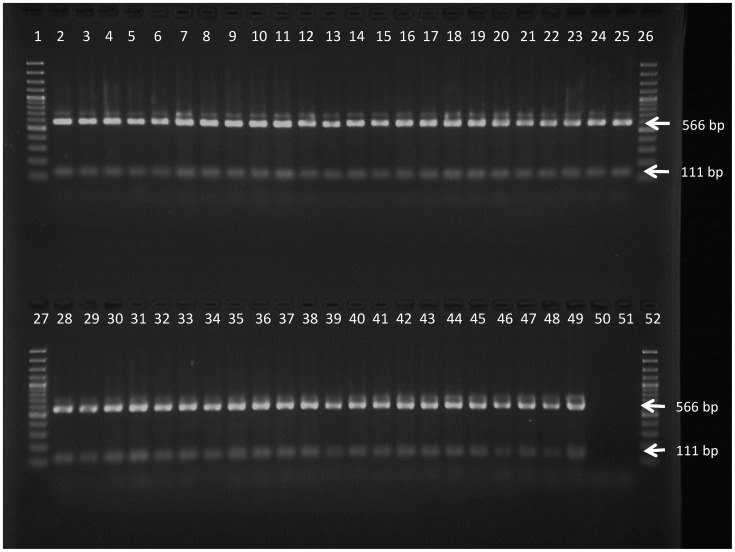
Amplicons generated with *TbAT1*-PCR on DNA of the *T.b. gambiense* strains as listed in Table 1 and of the two *T.b. brucei* control strains. Lanes 1, 26, 27 and 52 = GeneRuler 100 bp Plus DNA Ladder (Fermentas), lanes 2 to 44 = *T.b. gambiense* strains isolated from Mbuji-Mayi, lanes 45 to 48 = *T.b. gambiense* strains isolated from Masi-Manimba, lane 49 = *T.b. brucei* 427 WT, lane 50 = *T.b. brucei* 427 AT1/P2 KO, lane 51 = negative PCR control.

PCR with the Sfa primers yielded the expected 677 bp amplicon in all *T.b. gambiense* and in the *T.b. brucei* 427 WT strain but not in the *T.b. brucei* 427 AT1/P2 KO strain. Restriction enzyme digestion of the PCR amplicons with SfaNI showed the wild type pattern, i.e. one fragment of 566 bp and one of 111 bp, in all the *T.b. gambiense* and in the *T.b. brucei* 427 WT strains (Figure 1). With all the 45 *T.b. gambiense* strains and with the *T.b. brucei* 427 WT, the *TbAT1* PCR generated an amplicon of about 1600 bp, indicating the presence of the P2 adenosine transporter gene (Figure 2). Only the *T.b. brucei* 427 AT1/P2 KO strain yielded a fragment of about 2000 bp indicative of the replacement of P2 adenosine transporter gene by antibiotic resistance genes in the Tb*at1* null mutant. For selected strains the entire *TbAT1* gene was sequenced (Table 3). Twelve strains from Mbuji-Mayi and all four strains from Masi-Manimba had a *TbAT1* sequence identical to the wild-type *TbAT1* sequence of *T.b. gambiense* 930 (KF564940). However, two old Congolese *T.b. gambiense* strains, MBA and KEMLO, contained a not yet described trinucleotide deletion in *TbAT1*_Δ516–518_. Aquaglyceroporin transporters

**Table 3:** Genotype, strains and accession numbers for *TbAT1*.

Genotype	Strains	Accession number
*TbAT1*	15BT	KM282018
	45BT	KM282019
	108BT	KM282020
	108AT	KM282021
	130BT	KM282022
	146AT	KM282023
	163AT	KM282024
	174BT	KM282025
	174AT	KM282026
	346AT	KM282027
	348BT	KM282028
	378BT	KM282029
	MM01	KM282030
	MM03	KM282031
	MM05	KM282032
	MM06	KM282033
*TbAT1*_Δ516–518_	MBA	KM282016
	KEMLO	KM282017

**Figure 3:**
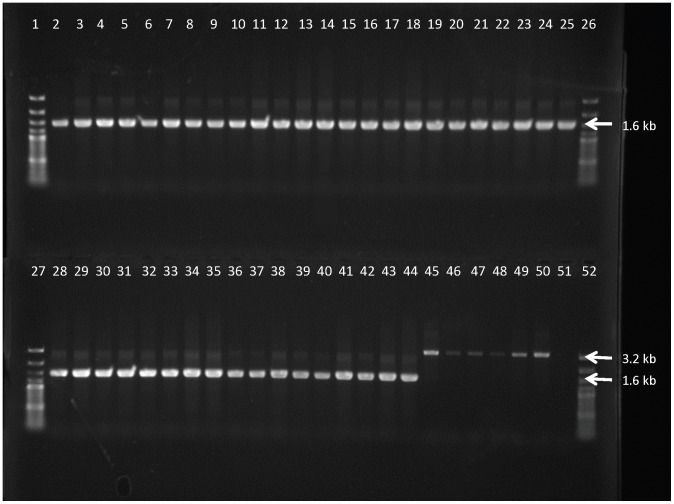
Amplicons generated with *AQP2/3*-PCR on DNA of the *T.b. gambiense* strains as listed in Table 1 and of the two *T.b. brucei* control strains. Lanes 1, 26, 27 and 52 = GeneRuler 100 bp Plus DNA Ladder (Fermentas), lanes 2 to 44 = *T.b. gambiense* strains isolated from Mbuji-Mayi, lanes 45–48 = *T.b. gambiense* strains isolated from Masi-Manimba, lane 49 = *T.b. brucei* 427 WT, lane 50 = *T.b. brucei* 427 AT1/P2 KO, lane 51 = negative PCR control.

**Figure 4:**
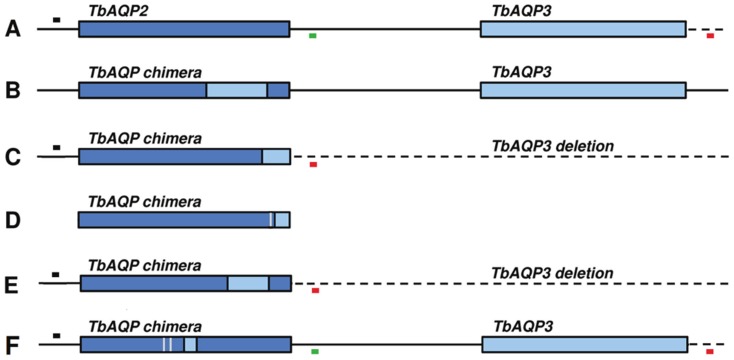
Schematic view of the *AQP2/3* variants identified in this study (adapted from Graf et al [20]). Sequence of *AQP3* was not verified. Positions of primer: black box = AQP2/3_F, green box = AQP2_R, red box = AQP2/3_R. A) Reference locus of *AQP2* and *AQP3*, with wild-type *AQP2* found in the melarsoprol and pentamidine sensitive strain *T.b. gambiense* LiTat 1.3 and in all strains from Masi-Manimba. B) Chimera of *AQP2* and *AQP3* occurring in a melarsoprol and pentamidine resistant *T.b. brucei* strain as described by Baker *et al.* [19]. C) Chimera of *AQP2* and *AQP3* plus loss of *AQP3* in all *T.b. gambiense* strains from Mbuji-Mayi as described in this article and by Graf et al. [20]. D) New chimera of *AQP2* and *AQP3*, possibly outside the known locus, found in all *T.b. gambiense* strains from Mbuji-Mayi. E) New chimera of *AQP2* and *AQP3* plus loss of *AQP3* found in two old Congolese *T.b. gambiense* strains, MBA and KEMLO. F) New chimera of *AQP2* and *AQP3*, without loss of *AQP3*, found in all four *T.b. gambiense* strains isolated in Masi-Manimba.

With all the 41 *T.b. gambiense* strains from Mbuji-Mayi and in both MBA and KEMLO, no amplification was seen when the wild type *AQP2* locus was amplified with an *AQP2* locus specific PCR. In contrast *AQP2/3*-PCR generated one single amplicon of about 1500 bp (Figure 3) previously shown to represent the chimeric *AQP2/3* gene and the deletion of the *AQP3* gene [20]. With the 4 *T.b. gambiense* strains from Masi-Manimba and the *T.b. brucei* 427 WT as well as the *T.b. brucei* 427 AT1/P2 KO, one single amplicon of about 1500 bp was found with the *AQP2* locus specific PCR, while in *AQP2/3*-PCR a band of 3200 bp was generated, indicating the presence of both the *AQP2* and the *AQP3* genes. Direct sequencing of the *AQP2*/3 amplicons of twelve of the Mbuji-Mayi strains showed that the band was indeed the previously described *AQP2*/3 chimera (Table 4), containing the first 813 bp from *AQP2* and the last 126 bp from *AQP3*, abbreviated here as *AQP2/3*_(814)_ to indicate that the switch from *AQP2* to *AQP3* is first detected at nucleotide 814 (Figure 4 – C). Amplicons from MBA and KEMLO, revealed a yet unknown *AQP2*/3 chimera, containing the first 677 bp from *AQP2*, followed by a stretch of 202 bp of *AQP3* and ending with the last 60 bp from *AQP2*, abbreviated here as *AQP2/3*_(678–880)_ to indicate that the switch from *AQP2* to *AQP3* is first detected at nucleotide 678 and the switch back to *AQP2* is first detected at nucleotide 880 (Figure 4 – E). Direct sequencing results from the circa 1500 bp *AQP2* amplicons from 4 strains from Masi-Manimba consistently showed a total of 18 heterozygous single nucleotide polymorphisms in comparison to the wild-type *AQP2* sequence of *T.b. gambiense* STIB 930 (KF564925). After cloning of the full length *AQP2* sequence from one strain of Masi-Manimba, MM01, we obtained either the wild-type sequence or another novel chimera that shares the first 616 bp from *AQP2*, with 2 point mutations at position T548C and G573A, followed by a stretch of 41 bp identical to *AQP3* and ending with the last 282 bp from *AQP2*, abbreviated here as *AQP2/3*_(617–658)_ (Figure 4 – F). Cloning *AQP2* sequences from LiTat 1.3, the West-African *T.b. gambiense* strain sensitive to melarsoprol and pentamidine, revealed only wild-type sequences (Figure 4 – A), while cloned *AQP2/3* sequences from the old *T.b. gambiense* MBA strain did not reveal any differences with results already obtained by direct sequencing (Figure 4 – E). Surprisingly, cloning of the *AQP2/3* gene from *T.b. gambiense* 348 BT revealed next to the chimera found by direct sequencing yet another *AQP2/3* chimera that shared the first 879 bp from *AQP2*, with one point mutation at T869C and only the last 60 bp from *AQP3*, abbreviated as AQP2/3_(880)_ (Figure 4 – D). A partial alignment of the obtained sequences of the *AQP2*, *AQP3* and *AQP2/3* variants is given in figure S1. When *T.b. gambiense* 348 BT was amplified with a cloning primer specific for *AQP2*, only a very faint amplification pattern was seen, probably attributable to primer mismatch on *AQP2/3* (figure S2).

**Table 4:** Genotype, strains and accession numbers for *TbAQP2* and *TbAQP2/3*.

Genotype	Strains	Accession number
AQP2	LiTat 1.3	KM282048
	MM01*	KM282049
AQP2/3 (814)	15BT	KM282036
	45BT	KM282037
	108BT	KM282038
	108AT	KM282039
	130BT	KM282040
	146AT	KM282041
	163AT	KM282042
	174BT	KM282043
	174AT	KM282044
	346AT	KM282045
	348BT	KM282046
	378BT	KM282047
AQP2/3 (880)	348BT	KM282050
AQP2/3 (678–880)	MBA	KM282034
	KEMLO	KM282035
AQP2/3 (617–658)	MM01*	KM282051

*direct sequencing results suggested 18 heterozygous single nucleotide polymorphisms in the *AQP2* coding sequence of *T.b. gambiense* MM01, MM03, MM05, and MM06.

#### RFLP to discriminate Mbuji-Mayi *AQP2/3* variants

**Figure 5:**
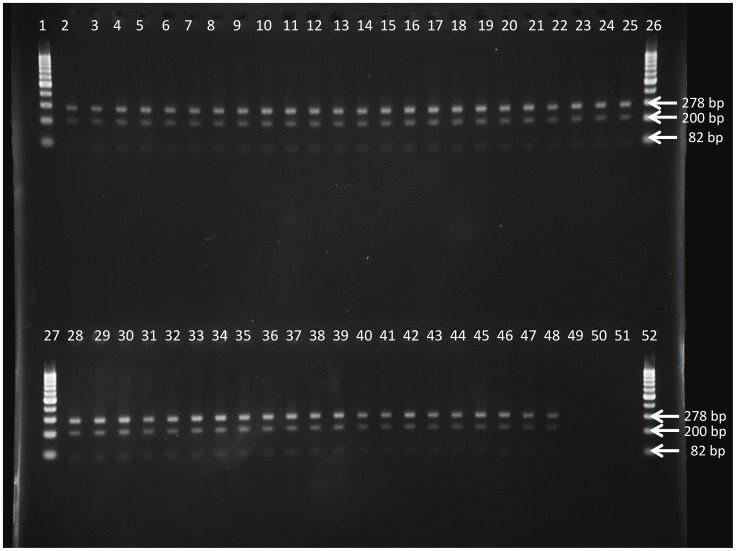
Restriction digest profile generated with AvaI PCR-RFLP on DNA of the *T.b. gambiense* strains as listed in Table 1, including the four strains isolated from relapsed mice. Lanes 1, 26, 27 and 52 = GeneRuler 100 bp Plus DNA Ladder (Fermentas), lanes 2 to 44 = *T.b. gambiense* strains isolated from Mbuji-Mayi, lane 45 = *T.b. gambiense* 15BT relapse 10 mg/kg BW, lane 46 = *T.b. gambiense* 163AT relapse 10 mg/kg BW, lane 47 = *T.b. gambiense* 346AT relapse 10 mg/kg BW, lane 48 = *T.b. gambiense* 346AT relapse 12 mg/kg BW, lane 49 = *T.b. gambiense* MBA, lane 50 = *T.b. gambiense* MM01, lane 51 = negative PCR control.

**Figure 6:**
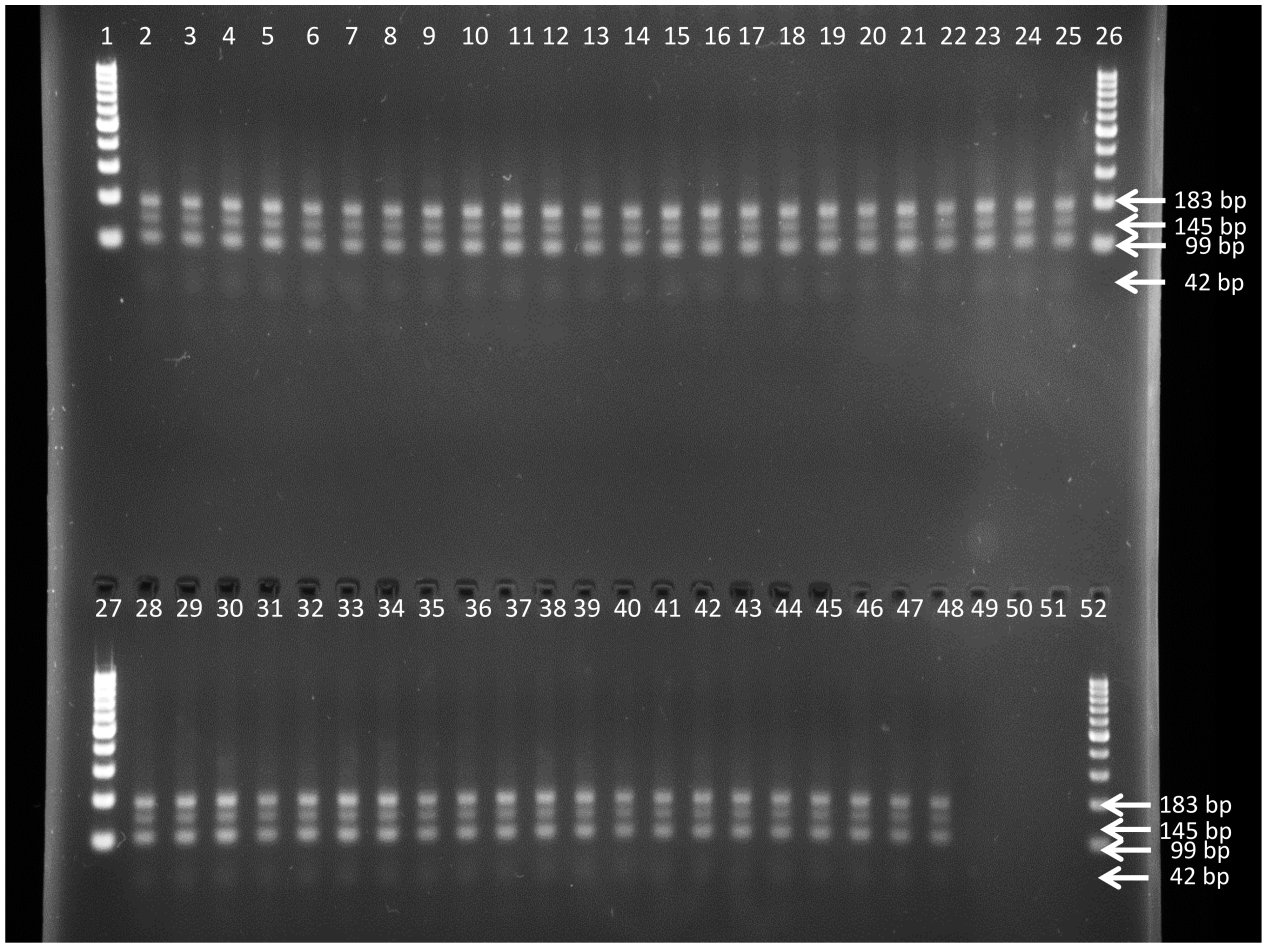
Restriction digest profile generated with SduI PCR-RFLP on DNA of the *T.b. gambiense* strains as listed in Table 1, including the four strains isolated from relapsed mice. Lanes 1, 26, 27 and 52 = GeneRuler 100 bp Plus DNA Ladder (Fermentas), lanes 2 to 44 = *T.b. gambiense* strains isolated from Mbuji-Mayi, lane 45 = *T.b. gambiense* 15BT relapse 10 mg/kg BW, lane 46 = *T.b. gambiense* 163AT relapse 10 mg/kg BW, lane 47 = *T.b. gambiense* 346AT relapse 10 mg/kg BW, lane 48 = *T.b. gambiense* 346AT relapse 12 mg/kg BW, lane 49 = *T.b. gambiense* MBA, lane 50 = *T.b. gambiense* MM01, lane 51 = negative PCR control.

Since *T.b. gambiense* 348 BT was isolated from a cured patient and seemed to contain both *AQP2/3*_(814)_ and *AQP2/3*_(880)_, we hypothesised that only *AQP2/3*_(814)_ would be present in strains isolated from relapsing patients. For this reason, we amplified a part of the *AQP2/3* gene using a PCR specific to the Mbuji-Mayi strains and used RFLP with either AvaI or SduI restriction enzymes to discriminate between both *AQP2/3* variants. RFLP with AvaI does not cut the *AQP2/3*_(814)_ chimera, but generates bands of 200 bp and 82 bp in the *AQP2/3*_(880)_ chimera. RFLP with SduI cuts *AQP2/3*_(814)_ chimera in bands of 99 bp and 183 bp, while *AQP2/3*_(880)_ chimera is cut in bands of 99 bp, 145 bp and 42 bp. PCR-RFLP on plasmids containing only a single *AQP2/3* variant could unambiguously differentiate between both *AQP2/3*_(814)_ and *AQP2/3*_(880)_ (figure S3). However, PCR-RFLP, with either AvaI (Figure 5) or SduI (Figure 6), on all *T.b. gambiense* strains from Mbuji-Mayi, including four strains isolated from relapses from mice, indicated the presence of both variants of the *AQP2/3* chimera, suggesting that strains that cause a relapse in either mice and humans are not genetically characterised by a loss of *AQP2/3*_(880)_.

## Discussion

This study was undertaken to investigate the mechanisms underlying the high relapse rates observed in second stage *gambiense* HAT patients treated with melarsoprol in Mbuji-Mayi, DRC. The *in vivo* melarsoprol sensitivity experiment showed that a minimum dose of 10 mg/kg BW melarsoprol was needed to cure mice infected with trypanosomes that were isolated from a cured patient. This is 4 times higher than the dose needed to cure mice infected with *T.b. gambiense* strains isolated from Ibba in South Sudan, another HAT focus known for high melarsoprol relapse rates [9]. On the other hand, Kibona and co-workers considered 3 out of 35 tested *T.b. rhodesiense* strains as resistant when mice relapsed after treatment with 5 mg/kg BW melarsoprol [30]. Among the 41 strains from Mbuji-Mayi, 2 induced infections that could not be cured with 12 mg/kg BW melarsoprol. Both were isolated from patients after treatment with melarsoprol.

When setting up the *in vivo* drug sensitivity experiment, we were confronted with the lack of a standardised protocol, especially for *T.b. gambiense*. Most studies have been dealing with *T.b. brucei* or *T.b. rhodesiense*, both behaving quite virulent in laboratory mice. Although melarsoprol is a drug that can cure the chronic phase of trypanosomosis, we opted for an acute phase *in vivo* model for several reasons: i. the concentration of melarsoprol that reaches the central nervous system is only a minor fraction of what reaches the plasma and is more prone to uncontrolled variations among individual outbred animals, ii. the acute phase model is expected to correspond better with the standard *in vitro* model, iii. since *T.b. gambiense* can cause subclinical or even silent infections, assessing treatment outcome in a chronic model via examination of blood and organs, including the brain, is unreliable [31]. The protocol we used here is mainly based on the study that Maina et al. carried out on recent isolates of *T.b. gambiense* from Sudan [9]. We also immunosuppressed the mice before inoculation with 5×10^4^ trypanosomes to guarantee that all mice would become infected. Some major differences however are to be noted. During the preparatory experiments, we noted that melarsoprol precipitates immediately when diluted in water, the usual diluent in other *in vitro* and *in vivo* studies [7], [9], [30]. In our final protocol, we diluted melarsoprol in polyethylene glycol to keep it in solution facilitating correct dosage when treating the mice. In contrast to the custom 60 days after treatment follow-up, we monitored the mice for up to 100 days after treatment and we immunosuppressed them on day 85 after treatment. In addition, at the end of the follow-up period, we sacrificed the mice and passed all the blood on DEAE cellulose columns instead of checking only a few drops of tail blood with the microhaematocrit technique. This allowed us to observe relapses at days 85–90 after treatment that otherwise would have been missed. Still, some treated mice showed paralysis but without any detectable trypanosome in the blood, suggesting that the real relapse rate was higher than what we actually can report based on trypanosome detection only. A weakness in our study is the absence of well documented *T.b. gambiense* melarsoprol resistant control strain. In the absence of such a strain, we had to rely on the *T.b. brucei* 427 AT1/P2 KO strain and its corresponding *T.b. brucei* 427 wild-type strain. Both strains appeared to be sensitive to melarsoprol at 10 mg/kg BW which is consistent with what has been observed in previous studies on a *Tbat1* null mutant [14]. On the other hand, for 5 strains included in our *in vivo* experiment, it was shown *in vitro* that they were 2–4 times less sensitive for melarsoprol than the reference sensitive *T.b. gambiense* strain STIB 930 [20]. Basing our treatment dose for the *in vivo* study solely on the dose required to cure a stabilate isolated from a cured patient proved to be a limitation for further interpretation of the *in vivo* results. Our initial hypothesis, including the rationale for the isolation of couples, was that strains originating from cured patients would be more sensitive to melarsoprol than strains from relapsed patients. It was clearly not expected that all strains would carry drug resistance markers. The different *in vivo* melarsoprol sensitivity phenotypes observed in our experiment do not correspond with the low variability observed within the two studied genetic markers associated with melarsoprol resistance. Indeed, all strains from Mbuji-Mayi, irrespective of their isolation from cured or relapsing patients, carry the wild type *TbAT1* allele. In addition, in all strains from Mbuji-Mayi, the *TbAQP2* and *TbAQP3* are replaced by chimeric *TbAQP2/3* variants, of which one has been reported previously to correlate with *in vitro* pentamidine and melarsoprol resistance by Graf and co-workers [20]. The latter study included 5 strains from the Mbuji-Mayi collection (40 AT, 45 BT, 130 BT, 349 BT, and 349 AT). According to Graf and co-workers, these 5 strains contained the *TbAQP2/3* chimera and the wild type *TbAT1*, what is confirmed in our study, and showed decreased sensitivity for pentamidine and for melarsoprol *in vitro*. In our study we found a second variant of a chimeric *AQP2/3* gene in these strains, which was only observed after cloning and not by direct sequencing, possibly indicating the presence of such variant outside the known *AQP2* locus. Surprisingly, the strains from Masi-Manimba were heterozygous for the *AQP2* locus. One allele contained the wild-type sequence, but the second allele contained a yet undescribed *TbAQP2/3* chimera. Munday and co-workers recently described that the presence of a functional wild-type *AQP2* sequence renders strains sensitive to melarsoprol and pentamidine [24]. However, the effect on pentamidine and melarsoprol uptake of the newly described *AQP2/3* chimeras is unknown. All variants were cloned in a trypanosomal expression vector for future evaluation and are available upon request. That this probable drug resistant genotype is found in all strains from Mbuji-Mayi and not in the strains from Masi-Manimba could be sufficient to explain the difference in melarsoprol relapse rates observed in East-Kasai (high) and in Bandundu (low). Within this context, it is interesting to note that we observed yet another *TbAQP2/3* chimera genotype in two “old” *T.b. gambiense* type I strains isolated in 1974 in Kinshasa and in Nord Equateur Province. Their *AQP2/3* variant is similar, but shorter, than the *AQP2/3* mutation described by Baker and co-workers and is therefore possibly capable of reducing melarsoprol and pentamidine uptake [19]. However, in contrast to the locus described by Baker and co-workers, *AQP3* seems not preserved in these strains. Both “old” isolates also contain a new variant of *TbAT1* with unknown effect on drug uptake. Due to the fact that both *AQP2/3* and *TbAT1* genes are different, these “old” isolates are probably not closely related to the contemporary strains circulating in Masi-Manimba and Mbuji-Mayi. Appearance of resistance to arsenicals, including melarsoprol, and to pentamidine in several HAT foci in DRC (former Congo Belge and Zaire) has already been described decades ago and was considered a result of mass treatment and chemoprophylaxis [32], [33]. The finding of the pentamidine/melarsoprol resistant genotype in the “old” *T.b. gambiense* strains may be the basis for molecular studies into the appearance and spread of pentamidine/melarsoprol resistant *T.b. gambiense* strains. In the present study, we didn't carry out microsatellite analysis to verify the similarity between strains from the Mbuji-Mayi focus, in particular from strains isolated from the same patient before treatment and after relapse. However, results from mobile genetic element PCR (MGE-PCR) as described by Simo *et al* suggest only very small differences between all strains from Mbuji-Mayi (figure S4) [34]. Thus, most probably, all strains isolated in Mbuji-Mayi are probably the clonal progeny of one strain acquiring the *AQP2/3* chimeras. This is the first large scale *in vivo* drug sensitivity study on *T.b. gambiense* strains that were isolated within a short period of time, in one single HAT focus, from cured as well as from relapsing patients and for 10 cases from the same patient before and after treatment. As such, the fact that the homogeneity observed within the *TbAQ2/3* and the *TbAT1* loci does not correspond with the heterogeneity in treatment outcome of the patients and of the mice, suggests that other factors, such as virulence and tissue tropism of the parasite or genotype and phenotype of the individual patient, may influence the treatment outcome. For example, in several independent studies it was found that high cell count in the cerebrospinal fluid (>100 cell/µl) is arisk factor for relapse [8], [23], [35]–[37]. This may indicate that patients in advanced second stage of the disease are less responsive for the standard melarsoprol treatment schedule. It is even not excluded that such patients are also less responsive for other drugs or therapeutic regimes such as NECT, the current first line treatment of second stage *gambiense* HAT. Therefore, further investigations into treatment failure in HAT and into alternative drugs or treatment regimes should not only focus on differential genotypes of the parasites but also on differential virulence and tissue tropism.

In conclusion, this study confirms that the high melarsoprol relapse rates observed in the Mbuji-Mayi focus can be explained by mutations in the *TbAQP2/3* locus of the trypanosomes that circulate in that focus. However, other factors will also influence the treatment outcome of individual patients.

## Supporting Information

Figure S1 Partial alignment of *AQP2*, *AQP3* and *AQP2/3* variants with GenBank accession numbers.
(TIF)

Figure S2 Amplification of *T.b. gambiense* DNA with a cloning primer specific for AQP2. Lanes 1 and 14: GeneRuler 100 bp Plus DNA Ladder (Fermentas), lanes 2–10: *T.b. gambiense* strains isolated from Mbuji-Mayi, lane 11 = *T.b. gambiense* LiTat 1.3, lane 12 = *T.b. gambiense* MM01, lane 13: negative PCR control.
(TIF)

Figure S3 RFLP with either AvaI or SduI restriction enzymes to discriminate between both AQP2/3 variants. RFLP with AvaI (panel A) does not cut the AQP2/3_(814)_ chimera (lane 1), but generates bands of 200 bp and 82 bp in the AQP2/3_(880)_ chimera (lane 2). RFLP with SduI (panel B) cuts AQP2/3_(814)_ chimera in bands of 99 bp and 183 bp (lane 4), while AQP2/3_(880)_ chimera is cut in bands of 99 bp, 145 bp and 42 bp (lane 5). Lanes 3 and 6: GeneRuler 100 bp Plus DNA Ladder (Fermentas).
(TIF)

Figure S4 Amplicons generated with mobile genetic element PCR (MGE-PCR) on DNA of diverse *T. brucei* strains. Lanes 1, 26, 27 and 51 = GeneRuler 100 bp Plus DNA Ladder (Fermentas), lanes 2 to 43 = *T.b. gambiense* strains isolated from Mbuji-Mayi, lanes 44 = *T.b. gambiense* MBA, lane 45: *T.b. gambiense* AnTat 22.1, lane 46 = *T.b. gambiense* AnTat 9.1, lane 47: *T.b. gambiense* LiTat 1.3, lane 48 = *T.b. rhodesiense* AnTat 12.1, lane 49: *T.b. rhodesiense* AnTat 25.1, lane 50 = *T.b. gambiense* type II Abba.
(TIF)
