# Supplementary figures and images for: Melarsoprol Sensitivity Profile of Trypanosoma brucei gambiense Isolates from Cured and Relapsed Sleeping Sickness Patients from the Democratic Republic of the Congo
Source: PLoS Negl Trop Dis. 2014 Oct 2;8(10):e3212. doi: 10.1371/journal.pntd.0003212 (PMC4183442; doi:10.1371/journal.pntd.0003212)

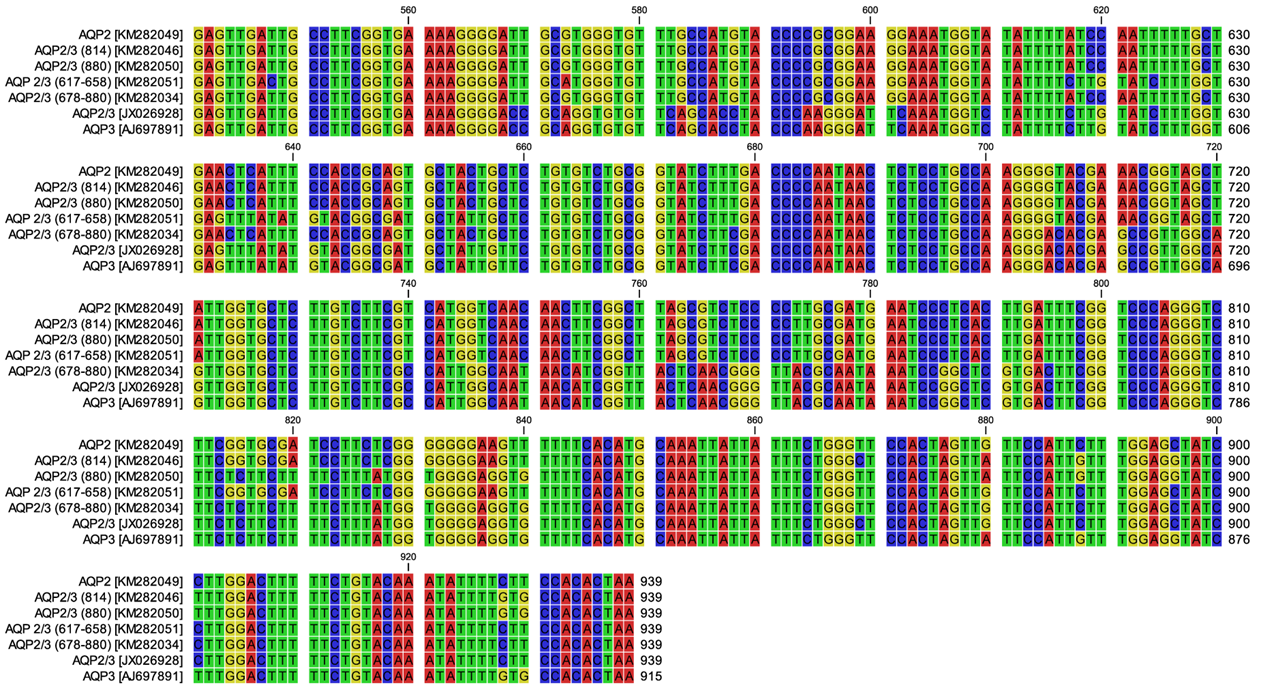

Supplement: Figure S1 — Partial alignment of AQP2, AQP3 and AQP2/3 variants with GenBank accession numbers. (TIF) [file pntd.0003212.s001.tif]
